# Detection of Mycobacteria in Arabian camels and antimycobacterial potential of *Moringa oleifera*

**DOI:** 10.1038/s41598-025-92402-0

**Published:** 2025-04-04

**Authors:** Sahar A. Allam, Eman Mahrous, Sahar T. M. Tolba, Samy M. Mohamed, Mohamed T. Ragab, Rania I. Mohamed

**Affiliations:** 1 Infectious Disease Unit, Animal and Poultry Health Department, Animal and Poultry Production Division, Desert Research Center, 1 Mataria Museum Street, Cairo, 11753 Egypt; 2 Technology Incubator for Nano Agricultural Applications, Desert Research Center, 1 Mataria Museum Street, Cairo, 11753 Egypt; 3 TB Unit, Bacteriology Department, Animal Health Research Institute, Agriculture Research Center, Giza, Egypt; 4 Microbiology Department, Faculty of Science, Ain Shams University, Cairo, Egypt; 5 Medicinal and Aromatic Plants Research Department, Pharmaceutical and Drug Industries Research Institute, National Research Center, Al-Buhouth Street, Dokki, Giza, Egypt; 6 Department of Pathology, Animal Health Research Institute, Mansoura Provincial Laboratory, Agricultural Research Center, Giza, Egypt

**Keywords:** Arabian camel, *Mycobacterium tuberculosis* variant *bovis*, Zoonosis, Infectious diseases, Sanger sequencing, Mi-seq Illumina, *Moringa oleifera*, GC–MS, Squalene, In vitro, In silico, Drug discovery, Genetics, Structural biology, Antimicrobials, Applied microbiology, Bacteriology, Clinical microbiology, Infectious-disease diagnostics, Microbiology, Bacteria, Infectious-disease epidemiology, Diseases, Infectious diseases, Tuberculosis, Sequencing, DNA sequencing, Next-generation sequencing, Phylogeny, Pathology, Plant sciences, Secondary metabolism

## Abstract

The World Health Organization gave great attention to *Mycobacterium tuberculosis*, especially its zoonotic impact. Dromedary camels in Arabian countries are of great importance, as well as awareness of production and health. Little was known about the occurrence of *M. tuberculosis* among Arabian camels. Out of 88 samples were collected from necropsied male camels aged 5–6.5 years after the slaughter process resident in Cairo abattoir. Isolation of Mycobacteria was achieved on Middle Brook 7H10 agar with special supplements, and then the suspected colonies were assessed by their specific aspects. Lungs and lymph nodes were processed for histopathology. Molecular characterization was carried out by both conventional amplification (*Mycobacterium bovis* mpb70, *M. tuberculosis*- Pan *Mycobacterium* 16S rRNA) tracked by sanger sequencing; and bacterial 16S rRNA V3–V4 hypervariable region was amplified then it was followed by Mi-seq Ilumina. *Moringa oliefera’s* oil was analyzed by GC–MS. The antimycobacterial potential of *M. oliefera* was conducted by In vitro tetrazolium microplate assay (TEMA). In silico docking mode of action and prediction were studied. *Mycobacterium* was isolated from 9.4% (3/32) of the lung samples and 2.4% (1/41) of the recovered lymph node samples. The isolated strains had ideal culture characteristics of *Mycobacterium*. Sanger sequencing identified the *M. tuberculosis* variant *bovis* DRC-EG-CAMEL PQ036932. Mi-seq Illumina revealed abundant sequence readings belonging to ancestral *Actinobacteria* and *Micromonosporaceae. *In vitro testing showed that the *Moringa oleifera* methanol leaf extract had antimicrobial activity with MIC ranging from 7.8 to 32 µg/ml, and the seed oil showed inhibitory effects at 50% (v/v) (*P* value < 0.05). In silico docking of ferulic acid against *M. tuberculosis* variant *bovis* ribosomal protein S1 showed an affinity score of − 5.95 kcal/mol with one hydrogen bond. While squalene lipoprotein LprF exhibited a professional affinity score of − 6.11 kcal/mol with seventeen hydrophobic π-interactions. *Mycobacterium tuberculosis* variant *bovis* is measured to prevail in the Arabian camels. However, this study provided a detailed examination of *Mycobacterium* in camels, offering practical solutions to combat this pathogen and mitigate the effects of infection or zoonotic impacts on other animals and humans. Sanger sequencing is more recommended for *Mycobacterium* identification. *Moringa oliefera’s* potential anti-mycobacterial effect through either leaves or oil might be achieved for humans and animals as a different strategy for medicinal plants’ role. It might be a new insight into the struggle and the adverse effects of tuberculosis. In the upcoming research, therapeutic compounds could be separated from *M. oliefera*.

## Introduction

**Fig. 1:**
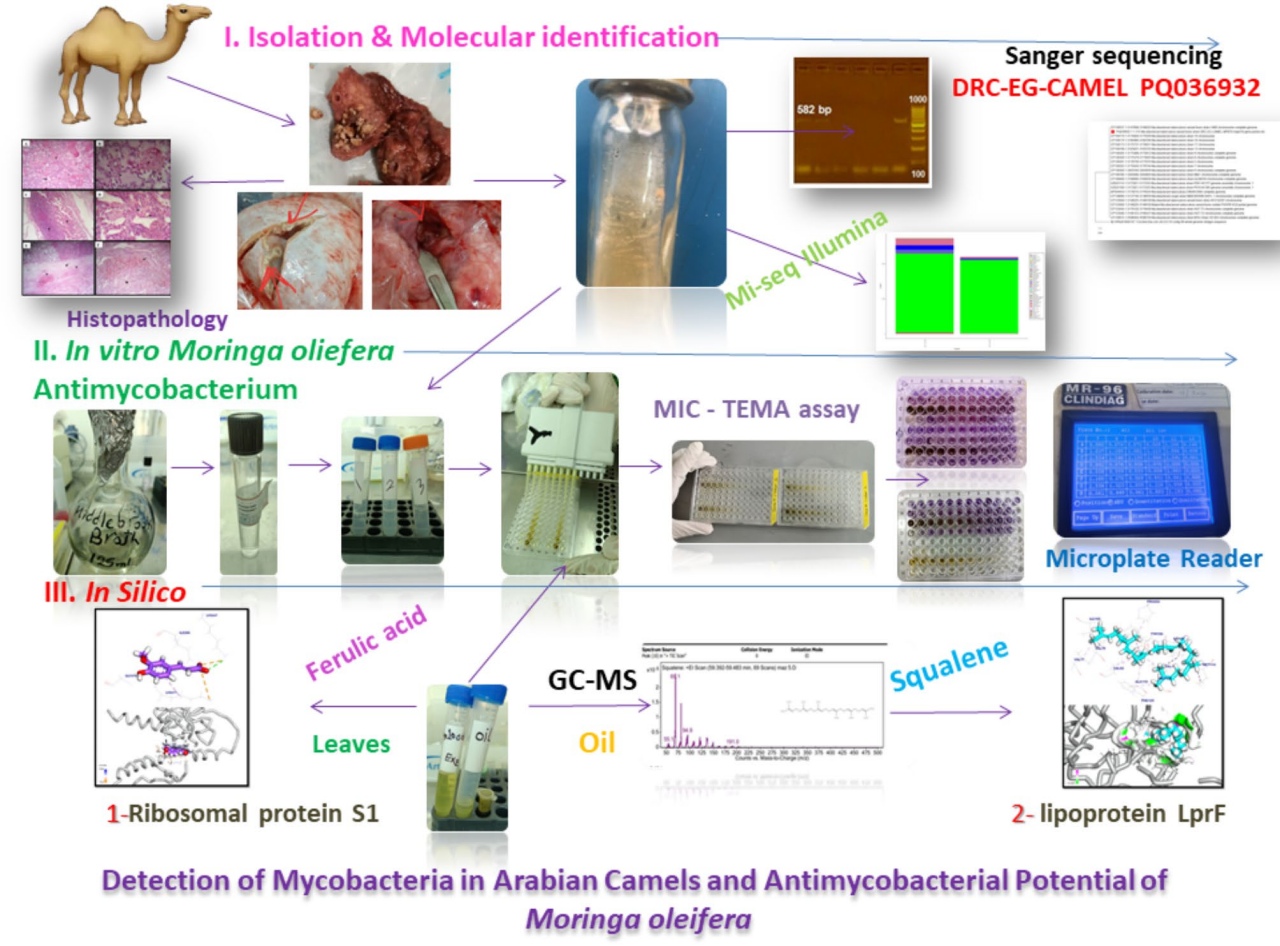
The collective schematic graphic abstract represents steps for Mycobacterial Isolation and identification from male camel with in vitro, in silico* Moringa oliefera* antimycobacterial potential.

Camels are of foremost importance in Arab and desert societies in terms of the inherited belief as a kind of sovereign wealth in these societies. Camels are considered a valuable source of meat, milk, and wool. Furthermore, they have cultural importance as they play a crucial role in sports competitions^1^. Therefore, we must care for the health of camels and maintain them, which is reflected in many of these aspects. Camels are characterized by their strong immunity^2^, which may cause the nature of some diseases to be unclear, which results in the spread of microbes during the incubation period of this pathogen, which may affect surrounding animals and humans. Among the diseases is tuberculosis in camels, which has not been addressed in detail before, and attempts to find solutions for them. The objective of this study is *Mycobacterium* isolation and identification from Arabian camels by two sequencing systems, Sanger and Mi-Seq Illumina, alongside in vitro anti-mycobacterial testing of *Moringa oleifera* oil and leaf extracts as a desert plant. Additionally, the study aims to predict the mode of action for active components in *M. oleifera* through in-silico bioinformatics. This study attempts to address the scientific gap that has not been discussed before to find natural solutions to limit the spread of *Mycobacterium* in camels and then try to control the zoonotic impact on humans. A collective schematic graphic abstract is represented in Fig. 1.

*Mycobacterium tuberculosis* is a species of pathogenic bacteria in the family *Mycobacteriaceae* and the etiology agent of tuberculosis^3^. Due to mycolic acid, *M. tuberculosis* has an unfamiliar, waxy coating on its cell surface. This coating makes the cells resistant to Gram staining and seems weakly Gram-positive^4^. Therefore, acid-fast stains such as Ziehl–Neelsen or fluorescent stains were used as an alternative to identify *M. tuberculosis* with a microscope. The physiological manner of *M. tuberculosis* is highly aerobic and obliges elevated oxygen levels. Mostly a pathogen of the mammalian respiratory system, it infects the lungs. The most often used diagnostic methods for tuberculosis are the tuberculin skin test, acid-fast stain, culture, and polymerase chain reaction^5–8^.

*Mycobacterium bovis* is a significant veterinary disease that infects cattle, causing bovine tuberculosis, which can spread to other species of mammals and humans through the inhalation of aerosols or the ingestion of unpasteurized dairy products^9,10^. *Mycobacterium bovis* is a zoonotic organism and, during the diagnostic examination, should be treated as a risk/hazard group III organism with appropriate precautions to prevent human infection from occurring^11–13^. *Mycobacterium bovis* and *Mycobacterium caprae* are considered members of the *M. tuberculosis* complex^14,15^. Transmission occurrence is due to aerosols as a principal factor: direct contact, food, and water^16^.

The Ministry of Health and Population in Egypt is making a significant effort to eliminate tuberculosis (TB) through many strategies, including early diagnosis. The gold standard method used in clinical mycobacterial laboratories is the culturing of clinical specimens obtained from the animal, as recommended by OIE in its last report in 2019^17^.

Sanger sequencing further assessment for identifying zoonotic and infectious bacteria identity was assigned for several years^18^. Next-generation sequencing (NGS) technology started a new era of genomic research^19,20^. The progressive stage of NGS is Mi-Seq Illumina, which has been reported to be more cost-effective and accurate for detecting the microbial community^21,22^. Metagenomic methods allow the investigation of entire bacterial microbiota associated with their vectors, allowing for better assessment of the diversity of going microbes and the reservoir potential of some vectors^23–25^. The NGS technique has some uses in veterinary applications^26–28^. Alteration of ruminant microbiomes alongside different physiological and nutritional stages has made this technique retain a pronounced role in this field of research^29,30^.

Natural plants have been extensively used for traditional medicine, which was preferable to ancient people. Low side effect and even rich polyphenol compounds retain their importance. Because of global antibiotic resistance, The World Health Organization endorses recommendations for this concern to encourage the use of medicinal plants^31^. In silico study and molecular docking have become essential analyses to confirm and predict the molecule-target interaction between suspected chemical compound drugs and molecular target receptors. It has enabled the virtual screening of loads of compounds in an inexpensive period^32^*.*

## Material and methods

### Ethics approval

This study follows the ethics guidelines of the Research Ethics Committee Desert Research Center, Egypt, and complied with relevant Egyptian legislation (Approval No. AH/NO. 2020-10-5). Informed consent was obtained from the Cairo Abattoir manager to use the slaughtered camel organs in this study.

### Collection of tuberculous lesions

**Fig. 2:**
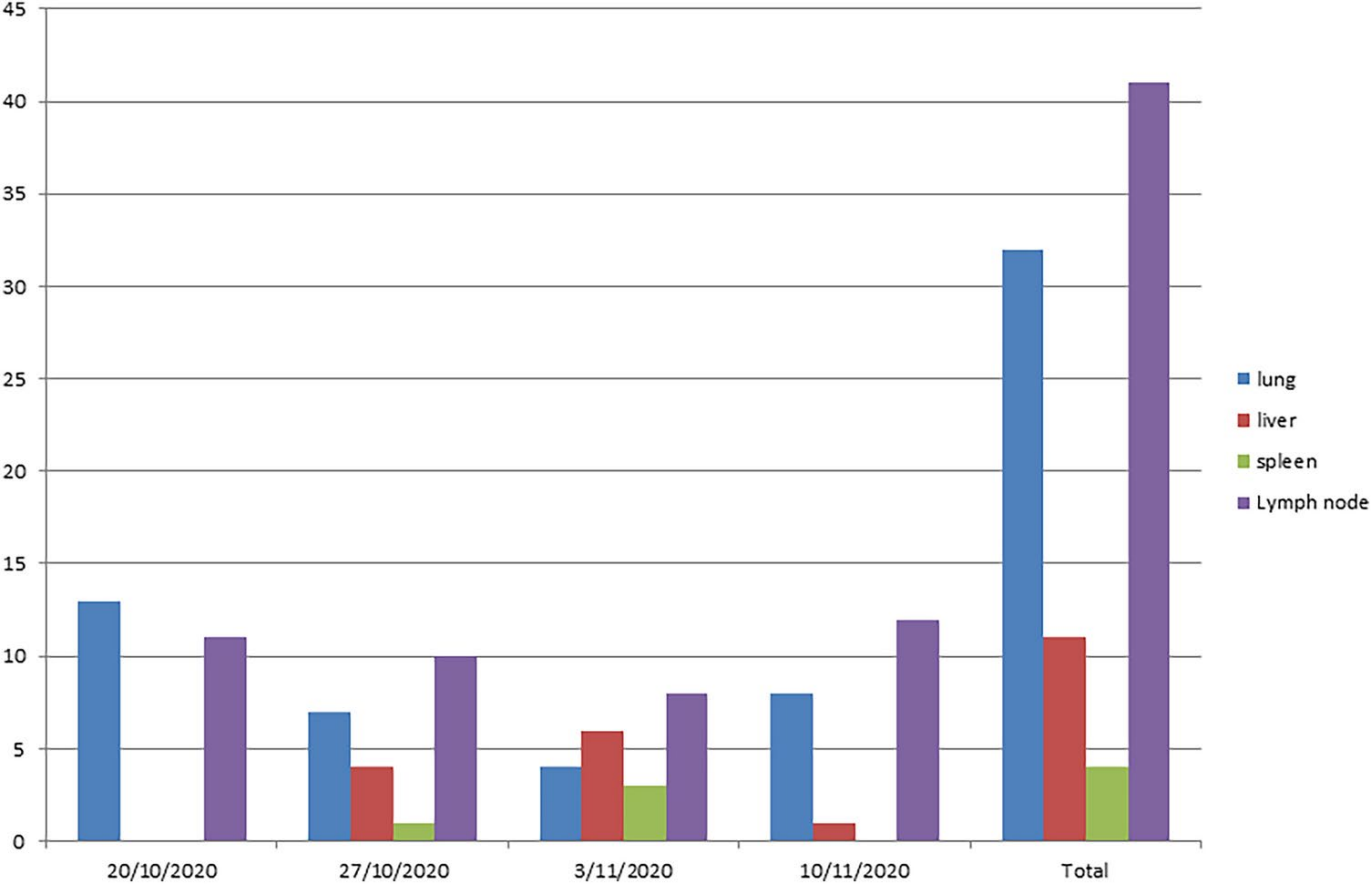
Periodic time for sample collection under study. Type, date, and number of the male camel organs from Cairo abattoir.

A total of 88 samples were collected from male camels aged 5–6.5 years after the slaughter process in an abattoir resident in Cairo. All camels came from Sudan and Somalia. Lesions in lymph nodes (particularly of the head and thorax), lungs, liver, and spleen samples were placed into sterile plastic bags for each sample. The samples were kept in an icebox with solid ice packs and transported to the Animal Health Research Institute TB unit for microbiological cultivation. The sample distribution is listed in Fig. 2.

### Ethical approval of the isolation protocol

The Microbiological procedures were conducted at the Bacteriology Department, TB unit, Animal Health Research Institute, Dokki, Giza, Egypt. The experiment protocol was approved by the Institutional Animal Care and Use Committee, Agriculture Research Center, Giza, Egypt.

### Sample processing and decontamination

Under the aseptic condition, the tissue specimen was transferred to a sterile mortar containing sterile sand. The fat was trimmed, and the suspected material was clipped into small pieces using sterile scissors and forceps; direct smears were made at this stage. The decontamination was performed as described by Sattar et al.^33^. Briefly, samples were inoculated in 0.9% hexadecylpyridinium chloride monohydrate (HPC) (Sigma-Aldrich, USA) and then were incubated at 37 °C for 24 h. The mixture was centrifuged at 10 °C (3000×*g*) for 15 min. The obtained pellets were re-suspended in 1 ml of sterile DW, then were mixed (using vortex/500 rpm for 30 s) with an equal volume of antibiotic suspension (vancomycin: 100 μg/ml, nalidixic acid: 100 μg/ml, and amphotericin: 50 μg/ml) (Oxoid, UK), followed by incubation at 37 °C for 24 h.

#### Isolation and identification

The processed samples were streaked on Middle Brook 7H10 agar (Oxoid, UK); media was supplemented by a 5 ml/l Glycerol (Oxoid, UK) and Middlebrook OADC Growth Supplement (Oxoid, UK). The inoculated plates were incubated at 37 °C (± 2 °C) under microaerophilic conditions where a CO_2_ sachet (Oxoid, UK) was placed in a tightly closed anaerobic jar. The incubated plates were examined for bacterial growth at 2, 4, 6, 8, 10, and 12 weeks (about three months) post-incubation. The suspected colonies were identified according to their culture characters, morphological characters using Ziehl–Neelsen staining, and biochemically using niacin production, nitrate reduction, tween-80 hydrolysis, thermo-stable catalase at 68 °C, and arylsulfatase tests as described by Quinne^34^.

#### Histopathological studies

Specimens from lungs and their neighboring lymph nodes were collected from necropsied camels and then fixed in 10% neutral buffered formalin solution, dehydrated in 70–100% gradual ethanol, and embedded in paraffin. Five-micrometer paraffin sections were prepared, stained with hematoxylin and eosin (H and E), and examined microscopically^35^.

#### Genetic characterization of camel mycobacteria

(I) Conventional polymerase chain reaction (PCR).

*DNA (Deoxyribonucleic acid) extraction.* DNA extraction from isolates was run by the QIA amp DNA Mini kit (Qiagen, Germany, GmbH). Briefly, 200 µl of the sample suspension was incubated with 10 µl (Microliter) of proteinase K and 200 µl of lysis buffer at 5 °C for 10 min. After incubation, 200 µl of 100% ethanol was added to the lysate. The sample was then washed and centrifuged according to the manual guide. Nucleic acid was eluted with 100 µl of elution buffer provided in the kit.

*Oligonucleotide Primer.* Primer sequences, according to Zhang et al.^36^, Tevere et al.^37^, and Quan et al.^38^, that were supplied from Metabion (Germany) are listed in Table 1.

**Table 1:** Primer sequences, target genes, product sizes, and cycling conditions used in PCR for *Mycobacterium.*

Target gene	Primers sequences 5′–3′	Amplified segment (bp)	Primary denaturation	Amplification (35 cycles)			Final extension	References
				Secondar denaturation	Annealing	Extension		
*Mycobacterium bovis* mpb70	**mpb70-N** F: ACCCTCAACAGCGGTCAGTAC	314	94 °C 5 min	94 °C 30 s	55 °C 40 s	72 °C 40 s	72 °C 10 min	Zhang et al.^36^
	**mpb70-C** R: TTACGCCGGAGGCATTAGCAC							
Pan *Mycobacterium* 16S rRNA	F:CACATGCAAGTCGAACGGAAAGG	582	94 °C 5 min	94 °C 30 s	62 °C 40 s	72 °C 45 s	72 °C 10 min	Tevere et al.^37^
	R: GCCCGTATCGCCCGCACGCTCACA							
*Mycobacterium bovis* (bovis-specific)	F CSB1: TTCCGAATCCCTTGTGA	255	95 °C 5 min	95 °C 45 s	57 °C 45 s	72 °C 1 min	72 °C for 10 min	Quan et al.^38^
	R CSB2:GGAGAGCGCCGTTGTA							
*Mycobacterium tuberculosis* (Tuberculosis-specific)	F CSB1: TTCCGAATCCCTTGTGA	575						
	RCSB3:AGTCGCGTGGCTTCTCTTTTA							

*PCR amplification.* Primers were utilized in a 25-µl reaction containing 12.5 µl of Emerald Amp Max PCR Master Mix (Takara, Japan), 1 µl of each primer of 20 pmol concentration, 5.5 µl of water, and 5 µl of DNA template. The reaction was run in an Applied biosystem 2720 thermal cycler.

*PCR product analysis.* The products of PCR were separated by electrophoresis on 1.5% agarose gel (AppliChem, Germany, GmbH) in 1 × TBE (TBE) buffer at room temperature using gradients of 5 V/cm. For gel analysis, 15 µl of the products were loaded in each gel slot. A ladder of 100 bp (Fermentas, Thermo, Germany) was used to determine the fragment sizes. A gel documentation system was used to photograph the gel (Alpha Innotech, Biometra).

(II) Sanger sequencing.

QIAquick PCR extraction kit was for PCR product purification (Qiagen, Valencia). A Perkin-Elmer sequencing kit was used, and then it was purified using a Centrisep spin column. DNA sequences were obtained by Applied Biosystems 3130 genetic analyzer (HITACHI, Japan). BLAST® analysis (Basic Local Alignment Search Tool)^39^ was initially performed to establish sequence identity to Gen Bank accessions. Mega11 was used for phylogenetic analysis^40^. The evolutionary history was inferred using the maximum likelihood method and the Tamura-Nei model^41^, with the bootstrap consensus tree by MEGA11^42^. Finally, Sanger sequencing was used to detect the exact species of *Mycobacterium* suspected isolates from Arabian camels.

(III) Mi-seq Ilumina-metagenome sequencing.

As previously mentioned, genomic DNA was extracted using the QIAamp DNA Mini kit (Qiagen, Germany, GmbH). We sent 10 gDNA (gDNA > 60 ng) to Macrogen, South Korea, for sequencing. Bacterial 16S rRNA V3–V4 hypervariable region was amplified from the extracted DNA using barcoded primers Bakt_341F: CCTACGGGNGGCWGCAG, Bakt_805R: GACTACHVGGGTATCTAATCC, https://www.macrogen.com. Paired-end sequencing (300 bp) was performed on the MiSeq platform (Illumina, Inc., San Diego, CA, USA) following the dual index sequencing strategy. Finally, Mi-seq Ilumina was conducted to predict Mycobacteria species using this modern technique.

#### *Moringa oliefera* analysis

(I) Moringa oliefera leaves analysis

We have previously prepared and analyzed *Moringa oliefera* leaves methanolic extraction by HPLC^43^.

(II) Moringa oliefera oli seed analysis by GC–MS (GC–MS QQQ 7890B GC system (USA)

*Moringa oleifera* oil seed extract was brought from the *Moringa* Society in the National Research Center, Giza, Egypt. Oil analysis was conducted at Cairo University-Faculty of Agriculture-Cairo Egypt (CURP) to discriminate the oil contents. Briefly, the control parameters were as follows: Oven equilibration time was 0.5 min, max temperature was 280 °C, oven program was 40 °C for 2 min, and then 4 °C/min to 280 °C for 2 min. Run time was 64 min for 2 min (Post Run) at 280 °C. Quench gas was Helium (He) at 2.25 ml/min, and collision gas was Nitrogen (N2) at 1.5 ml/min. The front injector syringe size was 10 µl, with an injection volume of 2 µl. GC–MS analysis was conducted using an Agilent column (19091S-433: 1. HP-5MS UI) at 325 °C, with dimensions of 30 m × 250 µm × 0.25 µm.

#### In vitro minimum inhibition concentration (MIC) detection of *Moringa oleifera *methanolic leaves and oil seed extracts against *Mycobacterium tuberculosis* variant *bovis* by tetrazolium microplate assay (TEMA)

Tetrazolium microplate assay TEMA was performed to detect the anti-mycobacterial potential as previously described^44,45^ with minor modifications. Briefly, 100 μL of Middle Brook 7H9 broth (pH 7.2; Sigma®, USA) was added to columns 2 to 11 in rows A to H, as labeled on the microtiter plates. One hundred microliters of 2 × concentration of amikacin was added to columns 1 and 2. The antibiotics were serially diluted twofold in consecutive columns by transferring 100 μl. The final drug concentrations in the wells were as follows: amikacin at 1 mg/ml, 1000, 500, 250, 125, 62.5, 31.25, 15.625, 7.8125, 3.9, 1.95, and 0.0976 μg/ml. The same procedure was performed for *M. oleifera*, methanol extract. For each isolate, 100 µl of 5mg/100 µl leaves extract, 100 μg/100 µl leaves extract, and oil were added to wells 1B, 1C, and 1D respectively.

The dilutions of the first leaf extract concentration began at 5, 2.5, 1.25, 0.625, 0.3125, 0.156, 0.78, 0.039, 0.0195, 0.000976, and 0.00488 mg/100 µl. The dilutions of the second concentration were started from 100, 50, 25, 12.5, 6.25, 3.125, 1.25, 0.78, 0.390, 0.195, and 0.0976 µg/100 µl. After filter-sterilization with 0.22 µm membrane, oil dilutions were 100, 50, 25, 12.5, 6.25, 3.125, 1.25, 0.78, 0.390, 0.195, and 0.0976 % (v/v). All methanolic and oil dilutions were performed in other rows that were kept as a control without microbial inoculation. A hundred microliters of mycobacterial suspension (set to McFarland Standard No.1) were added to wells in rows A to D in columns 1 to 11. The wells in column 11 serve as inoculum growth control without antibiotics. The plates were incubated at 37 °C with CO_2_ for five days. On day 5, 50 μl of the tetrazolium-Tween 80 solution was added to a11 wells except for isolate growth controls and extracts controls, and the plates were then incubated at 37 °C for 24 h. The plates were examined colourimetrically by a microplate reader (CLINDIAG Belgium) at 600 nm. A change in color from yellow to purple indicated the growth of bacteria, and the MICs were interpreted by comparing the readings of both tested and control wells. The lowest concentration at which there was no growth of *Mycobacterium* was taken as the minimum inhibitory concentration (MIC).

#### In silico molecular docking mode of action prediction of *Moringa oliefera*

The tested compounds were evaluated and screened against lipoprotein LprF and ribosomal protein S1 of *M. bovis* to predict the mode of action potential of *Moringa oliefera*. The 3D crystal structure of *M. bovis* target proteins was downloaded from the Protein Data Bank, http://www.rcsb.org/pdb. (PDBID: 4qa8: for LprF)^46^, and Uniprot ID: A0A0H3M6I9 https://www.uniprot.org/uniprotkb/A0A0H3M6I9/entry for ribosomal protein S1^47^. At first, the crystallized water molecules were removed from the downloaded crystal structure. Hydrogen atoms were added during molecular preparation, and energy minimization was carried out using the MMFF94 force field. Subsequently, hydrogen atoms were added to enhance clarity in the interaction areas. The 2D structures of the candidates were created using ChemBioDraw Ultra 14.0 and saved in the MDL-SD file format. Protonation of the tested compounds and energy minimization were performed using the MMFF94 force field. The docking process was done by Autodock Vina version 1.2.0, predicting approximately twenty poses. The optimal orientations were selected, and the 3D and 2D binding modes were generated using the Biovia Discovery Studio Visualizer. All relevant data were collected in Table 4.

### Statistical analysis

Statistical analysis (Microsoft Excel 2010) was applied by analysis of variance (ANOVA) single factor to evaluate the significant differences in probability value (*P* value < 0.05) between mean values of MIC of *Moringa oliefera* concentrations. Metagenomic analysis for NGS outcome was carried out through QIIME version 1.9.1. The parameter values were alpha diversity, Shannon, Chao1, Simpson, and observed species.

## Results

### Isolation

**Fig. 3:**
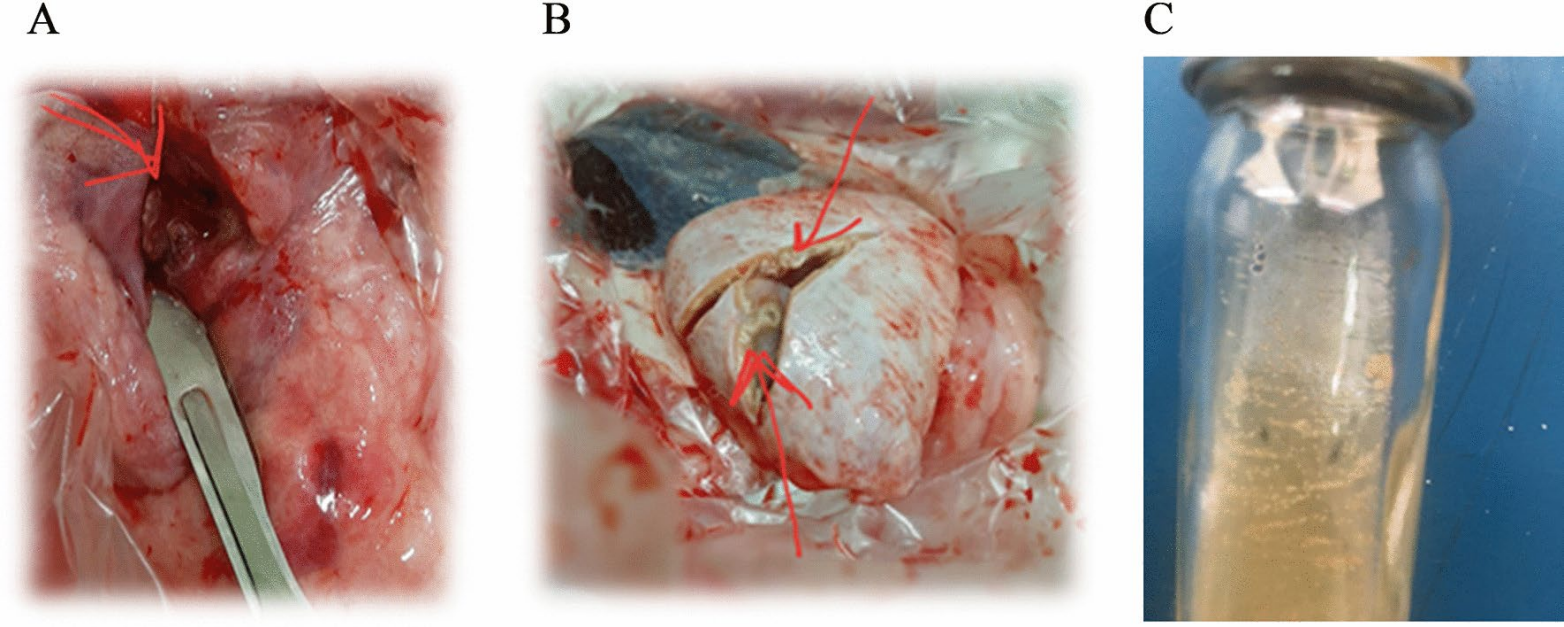
(**A**) lung and (**B**) lymph node suspected lesions with stone texture in male camel. (**C**) *Mycobacterium* buffed colonies after 60 days (about 2 months) of incubation at 37 °C with microaerophilic incubation.

**Fig. 4:**
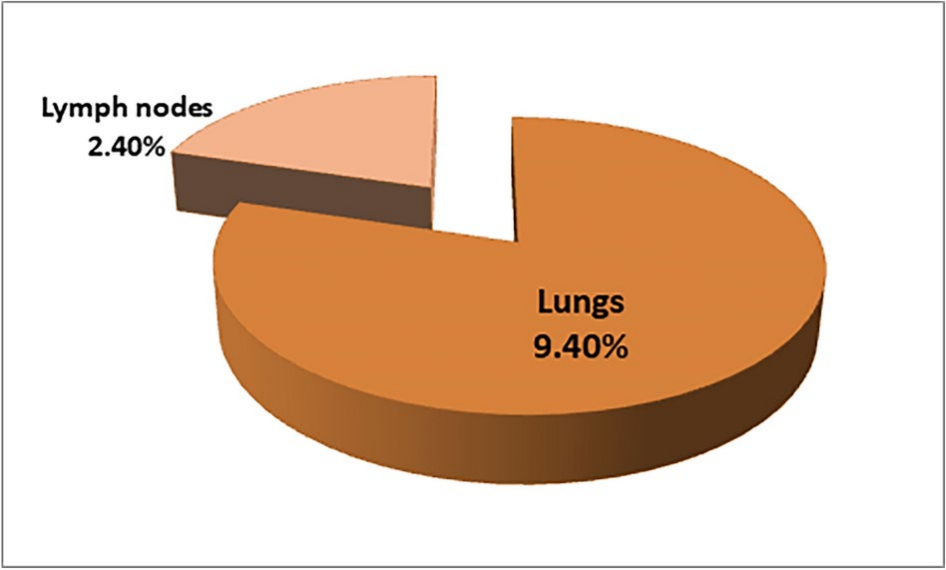
*Mycobacterium* isolation from lungs and lymph nodes of Arabian camels.

Four isolates, 4.5% (4/88), were obtained. However, the isolation from the lungs was 9.4% (3/32), and lymph nodes recovered 2.4% (1/41). The liver and spleen were in negative isolation. The isolates emerged after two full months in Middle Brook medium with microaerophilic incubation conditions of 37 °C (Figs. 3, 4).

#### Histopathology

**Fig. 5:**
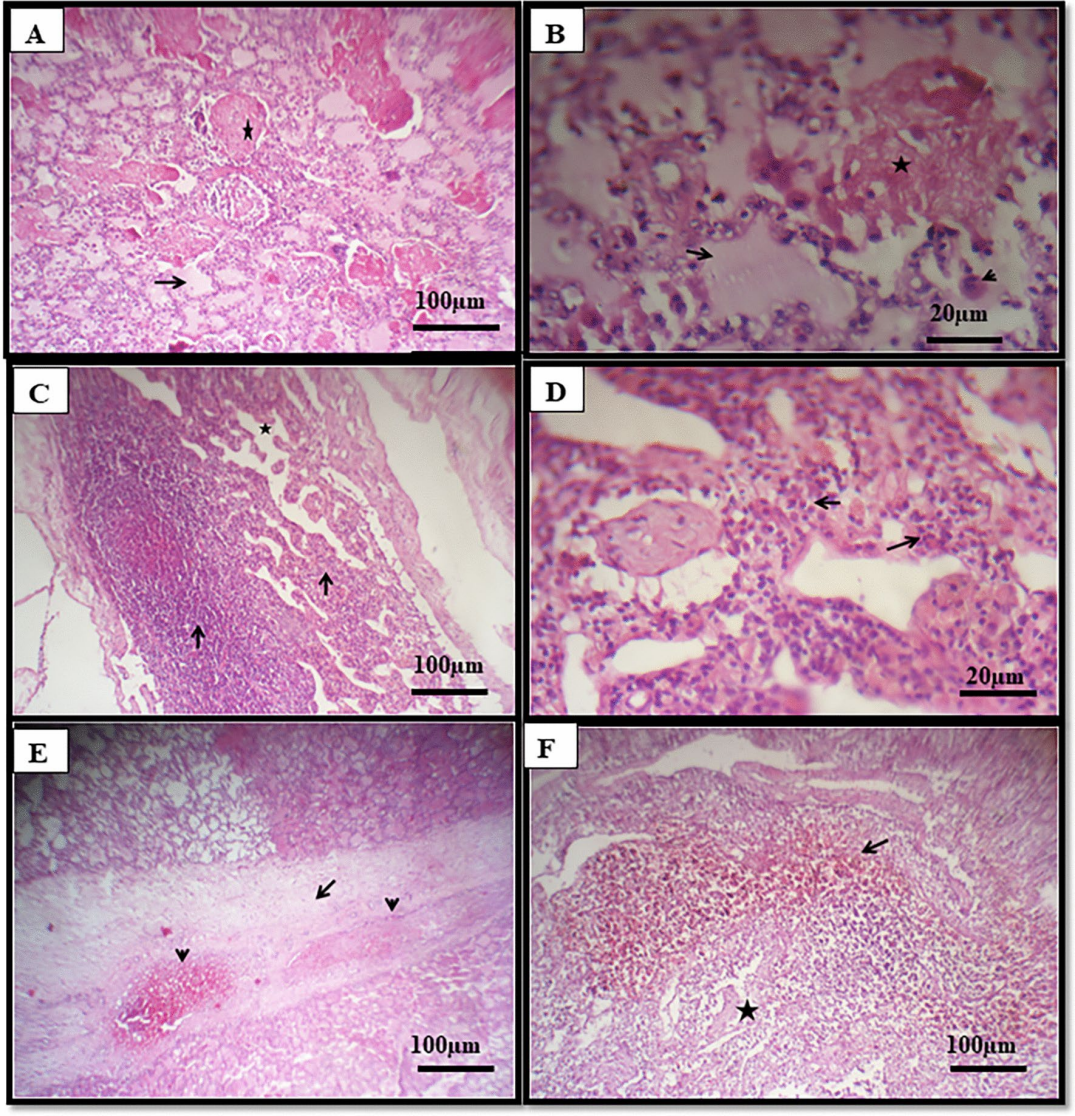
Histopathological examination. (**A**) The lung of the camel showed serous (arrow) and fibrinous (star) exudate within the alveoli (H&E); (**B**) High power of the previous figure to show serous (arrow) and fibrinous (star) exudate within alveoli beside heart failure cells (arrowhead) (H&E); (**C**) Lung of camel showing focal replacement of lung by lymphocytes and neutrophils (arrow) with thickening of interalveolar septae and atelectasis (star) (H&E); (**D**) High power of the previous figure to show thickened interalveolar septae by intense neutrophils (arrow) and lymphocytes (H&E); (**E**) Lung of camel showing thickening of pulmonary septae (arrow) by serofibrinous exudates and thrombosis of blood vessels (arrowhead) (H&E); (**F**) Lymph node of camel showing hemorrhages and hemosiderosis (arrow) and depleted follicles (star) (H&E).

The pulmonary lesions varied from distinct types of pneumonia, mainly serofibrinous, lymphocytic, and interstitial. The serofibrinous type was represented by extensive serous and fibrinous exudate within the alveoli, accompanied by heart failure cells (Fig. 5A,B). Lymphocytic pneumonia was characterized by focal replacement of lung tissue and interalveolar septae thickening by lymphocytes with or without neutrophils beside atelectatic alveoli (Fig. 5C,D). Pulmonary septae thickened by serofibrinous exudate with thrombosis of other blood vessels (Fig. 5E). Bronchial lymph node showed depleted follicles in both cortex and medulla with hemorrhage and hemosiderosis (Fig. 5F).

### Molecular characterization

(I) Conventional PCR for *M. tuberculosis* complex detection.

Screening four isolates by Pan *Mycobacterium* revealed two positive isolates at 582 bp, while the four isolates were positive for *M. bovis* at 314 bp, and three isolates were positive for *M. bovis* at 229 bp. All isolates were negative for 262 bp *M. tuberculosis*.

(II) Sanger sequencing.

**Fig. 6:**
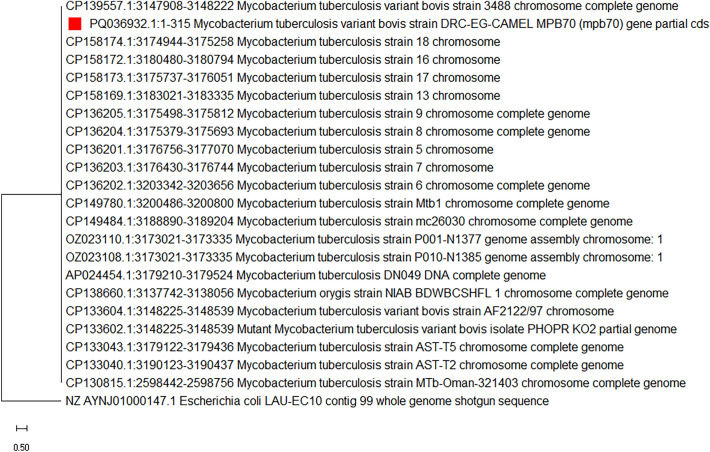
Evolutionary analysis was conducted for PQ036932 *Mycobacterium tuberculosis* variant *bovis* strain DRC-EG-CAMEL MPB70 (mpb70) gene, partial CDS by maximum likelihood method (Phylum: Actinomycetota, Class: Actinomycetia, Order: Mycobacteriales, Family: *Mycobacteriaceae*, Genus: *Mycobacterium*, Species: *M. tuberculosis*. The tree with the highest log likelihood (− 2606.31) is shown. The percentage of trees in which the associated taxa clustered is shown next to the branches. Initial tree(s) for the heuristic search were obtained automatically by applying Neighbor-Join and BioNJ algorithms to a matrix of estimated pairwise distances using the Tamura-Nei model, and then the topology with superior log likelihood value was selected. This analysis involved 23 nucleotide sequences. There were a total of 1493 positions in the final dataset. Evolutionary analyses were conducted in MEGA11.

Sequence analysis for *Mycobacterium* strain DRC-Eg-Camel was placed in the National Center for Biotechnology Information (NCBI) with accession number PQ036932. Evolutionary analysis was conducted for PQ036932 *M. tuberculosis* variant bovis strain DRC-EG-CAMEL MPB70 (mpb70) gene, partial CDS by maximum likelihood method. The tree with the highest log likelihood (− 2606.31) is shown in Fig. 6. Evolutionary analyses were conducted in MEGA11.

(III) Mi-seq Illumina.

**Fig. 7:**
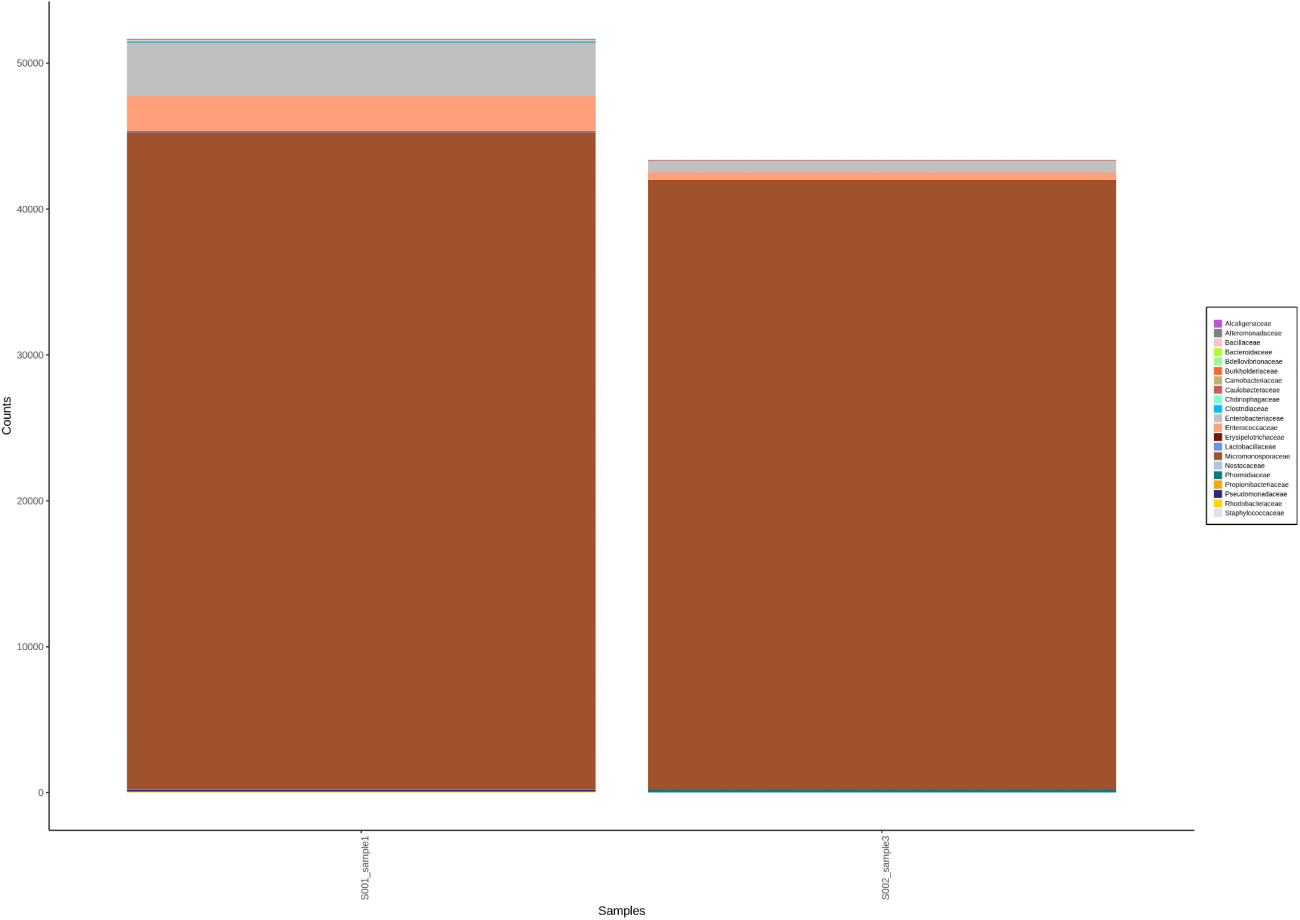
Screening for two isolates by OTU (Operational Taxonomy Unit) analysis to determine the family of isolates yielded abundant sequence reads of *Micromonosporaceae*.

**Fig. 8:**
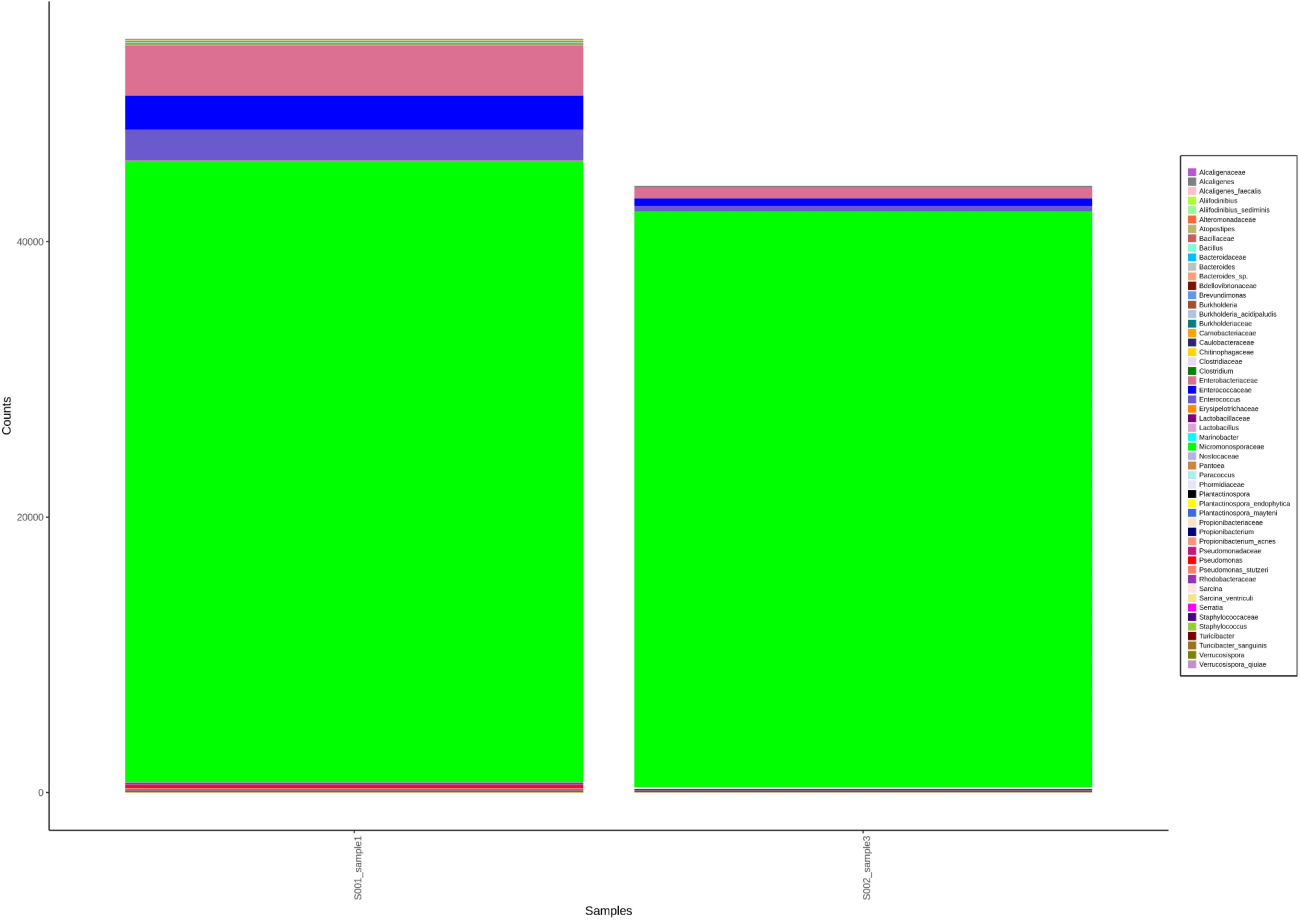
Screening for two isolates by OTU (Operational Taxonomy Unit) analysis to determine the family-genus-species of isolates yielded abundant sequence reads of *Micromonosporaceae*.

**Fig. 9:**
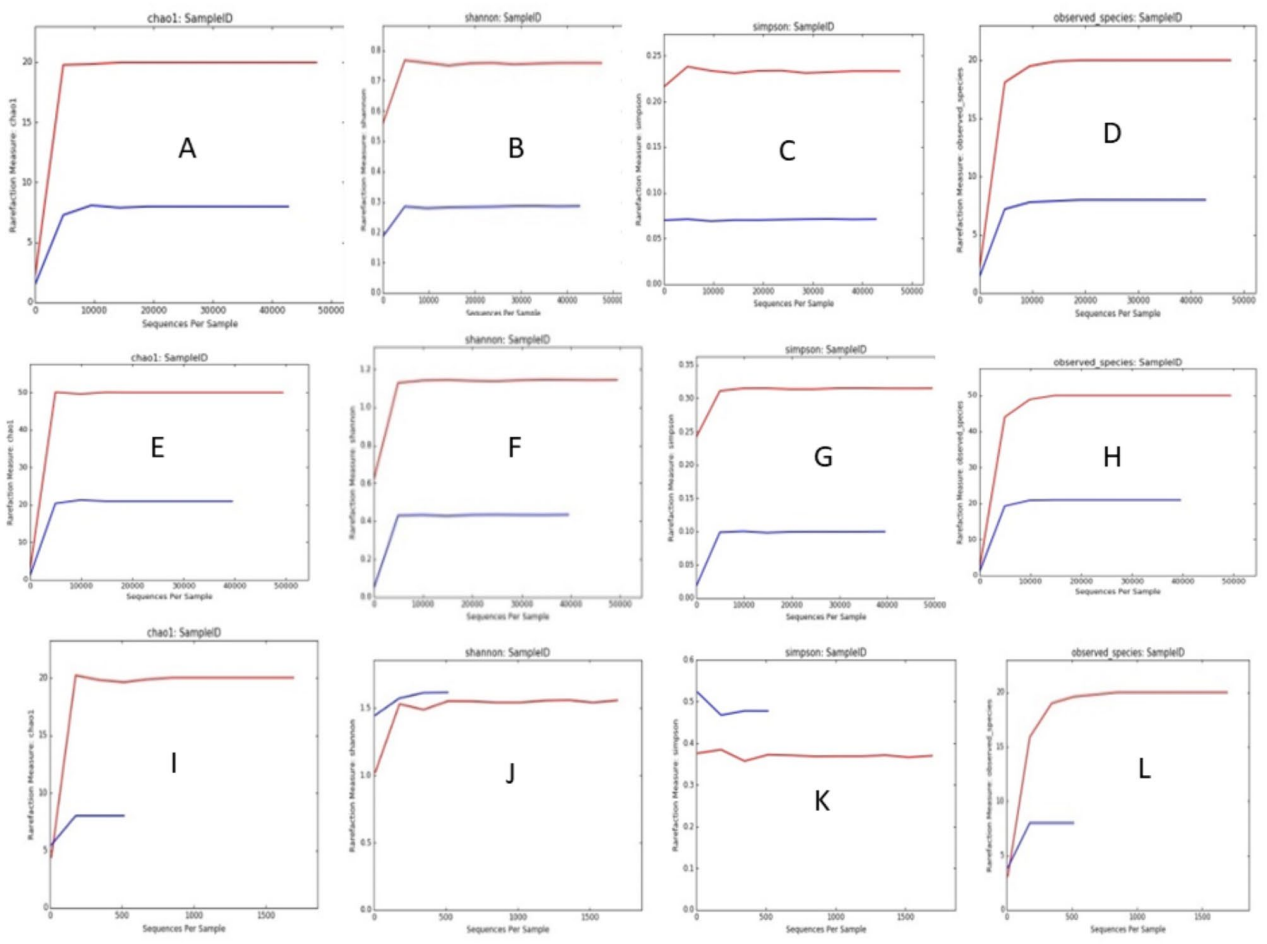
Parameter values for metagenomic analysis alpha diversity metrics within each of the two selected *Mycobacterium* isolates. The observed species (Y-axis) was limited after a certain number of sequence reads (X-axis) revealed a plateau (fixed sequence for *Micromonosporaceae*), which was shown in Chao, Simpson, Shannon, and alpha diversity for family (**A**–**D**), Alpha diversity for family, genus, and species (**E**–**H**), and Alpha diversity for genus (**I**–**L**).

Sequencing of the V3 and V4 regions of the 16S rRNA genes of *Mycobacterium* two isolates by Mi-seq Illumina are shown in Table 2. The total number of read sequences for the first isolate was 44,525,726 bp, the average reads were 174,926, the GC content (%) was 58.16%, and Q30% was 85.16%. The second isolate’s total read was 38,042,186 bp, the average read was 126,386, the GC content (%) was 59.2% and Q30% was 84.96%. AT% was 41.24% and 40.8%, while Q20% was 93.2 and 92.79% for the first and second isolates, respectively. Parameter values for metagenomic analysis alpha diversity metrics after Shannon, Chao1, Simpson, and observed species. Screening for two isolates by OTU (Operational Taxonomy Unit) analysis to determine the family of isolates yielded abundant sequence reads of *Micromonosporaceae*. The observed species were limited after a certain number of sequence reads showed a plateau (fixed sequence for *Micromonosporaceae*), which was shown in Chao, Simpson, Shannon, and alpha diversity for family, genus, and species (Figs. 7, 8, 9).

**Table 2:** Sequencing of the V3 and V4 regions of the 16S rRNA genes of two isolates of *Mycobacterium* by Mi-seq illumina.

Isolate	Read (bp)	Average reads	GC (%)	Q30%	Q20%	AT (%)
1	44,525,726	174,926	58.16	85.16%	93.2%	41.24
2	38,042,186	126,386	59.2	84.96%	92.79%	40.8

#### Gas chromatography-mass spectroscopy (GC–MS) analysis of *Moringa oleifera* oil

**Fig. 10:**
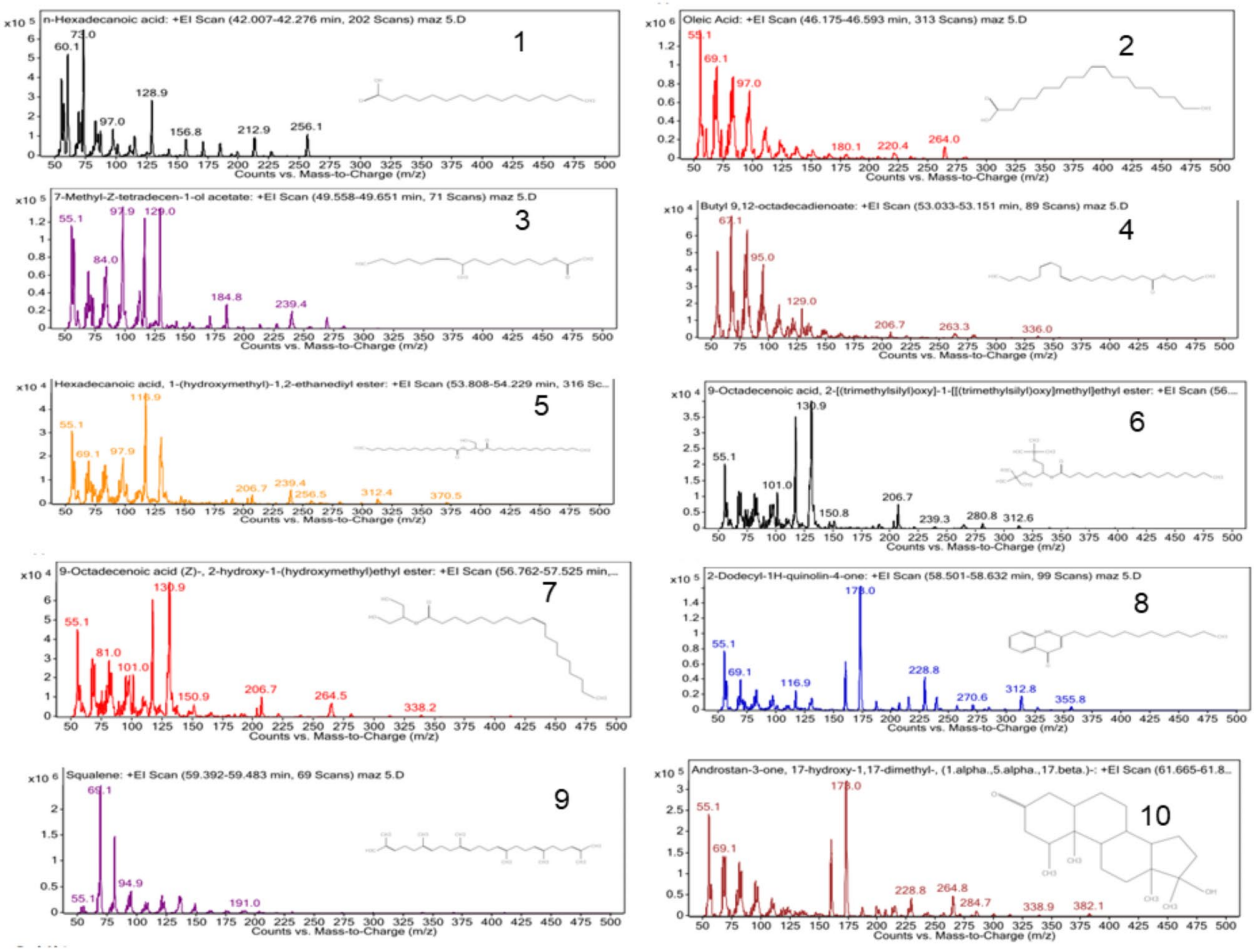
Qualitative analysis of *Moringa oleifera* oil seed by GC–MS. The peaks of each structure are as follows: seven essential oils were identified from the analysis, including hexadecanoic acid, oleic acid, 7 methyl-Z-tetradecene-1-ol acetate, butyl 9,12-octadecadienoate, with a percentage of 12.93%, 62.45%, 1.41%, 0.77%, peaks 1,2, 3 and 4 respectively, among the injected samples. Additionally, hexadecanoic acid 1-(hydroxymethyl)-1,2 ethandiylester, 9-Octadecenoic acid, 2-[(trimethylsilyl)oxy]1–1[[trimethylsilyl)oxy]methyl]ethylester, and 9-octadecenoic acid (Z)-2-hydroxy-1-(hydroxymethyl) ethylester were present at percentages of 1.54%, 1.24%, 4.59%, peaks 5,6, and 7, respectively. One compound as separate quinolone was 2-dodecyle-1H-quinolin-4-one 1.16% of 2 µl of the oil sample, peak 8. Finally, One hydrocarbon compound identified was squalene, peak 9 constituting 8.5% of the total volume, and an androstane structure accounted for 4.68% of the total volume in 2 µL of the injected oil sample, peak 10.

Seven essential oils were identified from the analysis, including hexadecanoic acid, oleic acid, 7 methyl-Z-tetradecene-1-ol acetate, butyl 9,12-octadecadienoate, with a percentage of 12.93%, 62.45%, 1.41%, 0.77%, respectively, among the injected samples. Additionally, hexadecanoic acid 1-(hydroxymethyl)-1,2 ethandiylester, 9-Octadecenoic acid, 2-[(trimethylsilyl)oxy]1–1[[trimethylsilyl)oxy]methyl]ethylester, and 9-octadecenoic acid (Z)-2-hydroxy-1-(hydroxymethyl) ethylester were present at percentages of 1.54%, 1.24%, 4.59%, respectively. One compound as separate quinolone was 2-dodecyle-1H-quinolin-4-one 1.16% of 2µl of the oil sample. Finally, One hydrocarbon compound identified was squalene, constituting 8.5% of the total volume, and an androstane structure accounted for 4.68% of the total volume in 2 µL of the injected oil sample. The peaks of each structure are shown in Fig. 10.

#### In vitro* Moringa oleifera* anti-mycobacterial by tetrazolium microplate assay (TEMA)

**Fig. 11:**
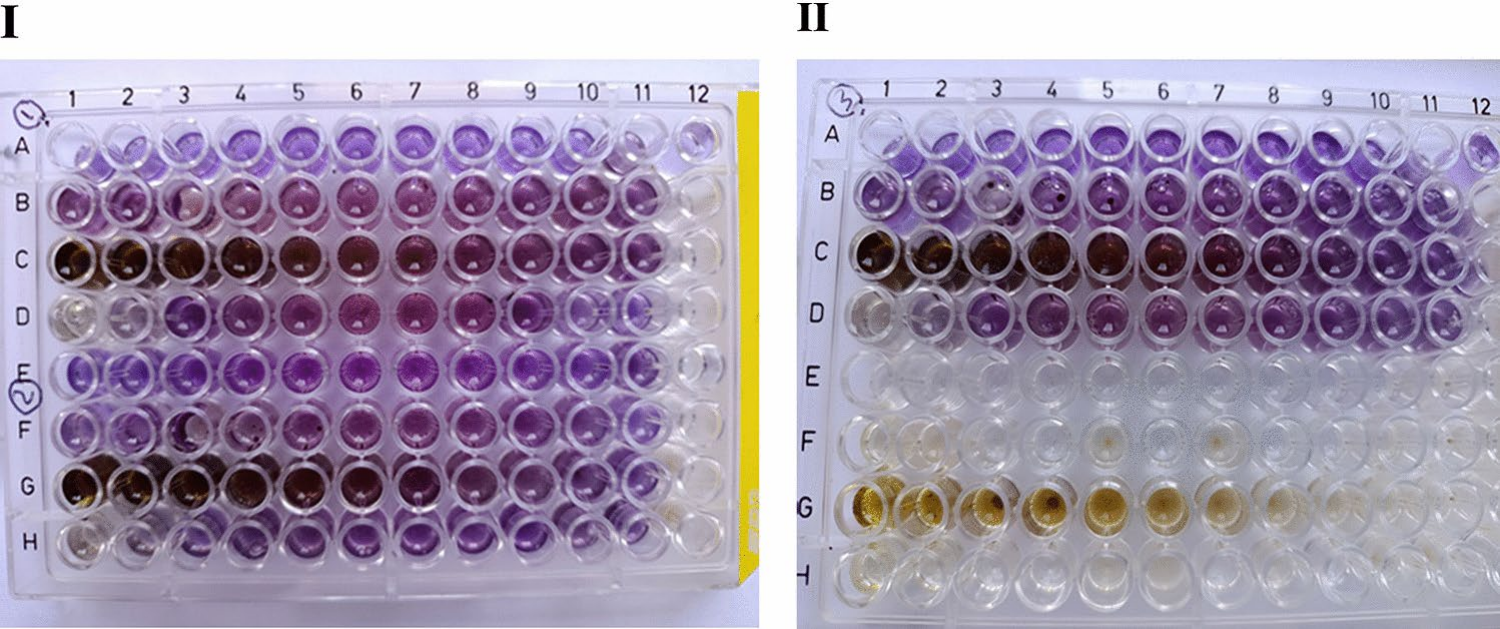
Minimum inhibition concentration (MIC) of *Moringa oleifera* by TEMA assay. (**I**) Rows A1–12 were amikacin dilutions with isolate1, B1 to 11 were dilutions of 100 µg/100 µl leaf extract, C1 to 11 were 5 mg/100 µl leaf extract, and D1 to D11 were oil dilutions. B12; C12 isolate 1 control (isolate growth with middle brook broth only). E1 to E12 were amikacin dilutions with isolate 2, F1 to11 were dilutions of 100 µg/100 µl leaves extract, G1 to11 were 5 mg/100 µl leaves extract, and H 1 to 11 were oil dilutions. F12, and G12 were isolate 2 control (isolate growth with middle brook broth only). (**II**) A1 to 12 were amikacin dilutions with isolate 3. B1 to11 were dilutions of 100 µg/100 µl leaf extract, C1 to11 were 5 mg/100 µl leaf extract, and D1 to D11 were oil dilutions. B12 and C12 were the control of isolate 3 (isolate growth with middle brook broth only). E1 to E12 were amikacin control dilutions, F1 to 11 were dilution controls of 100 µg/100 µl leaf extract, G1 to11 were dilution controls of 5 mg/100 µl leaves extract, H 1 to 11 were control oil dilutions. All controls were without microbial inoculations.

**Fig. 12:**
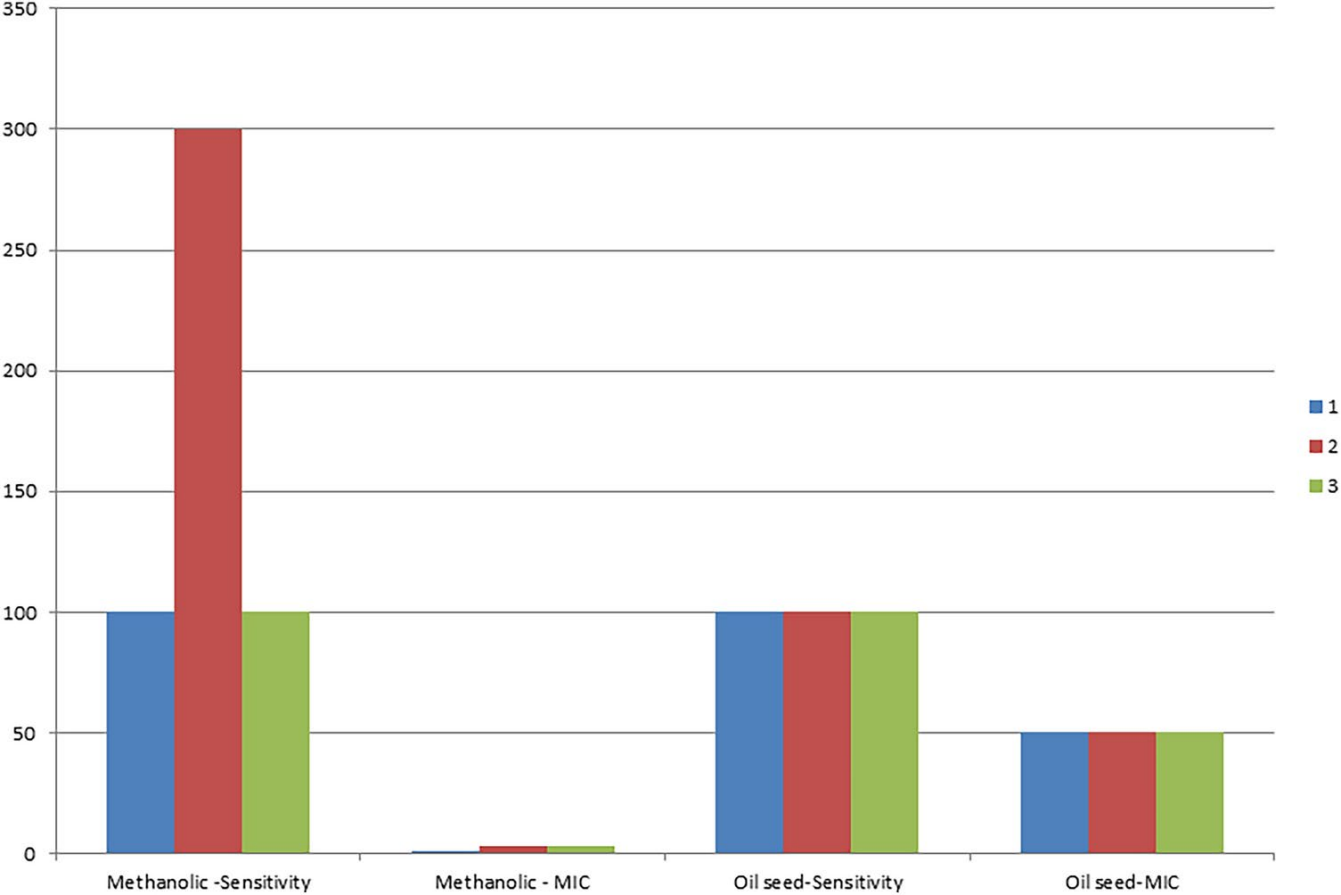
Sensitivity and minimum inhibition concentration (MIC) for both methanolic leaves (µg/100 µl) and oil seed extracts (v/v) of *Moringa oleifera* against *Mycobacterium tuberculosis* variant *bovis*. One, 2, and 3 are the *Mycobacterium* isolates.

The anti-mycobacterial activity was conducted on three isolates in duplicate for each; however, the test was started with two different concentrations from methanol extract of *M. oleifera*: 50 mg/ml and 1000 µg/ml. Each concentration is serially diluted. Oil started from 100%. Different inhibition reactions were among the three isolates, isolate number one sensitivity started from 1000 µg/ml, and MIC was 7.8 µg/ml. For isolate number two, sensitivity began from 3000 µg/ml, and MIC was equal to 32 µg/ml. Isolate number 3 sensitivity was initiated at 1000 µg/ml with MIC equal to 30 µg/ml. On the other hand, all isolates were inhibited by oil at 100% with MIC equal to 50% (v/v) dilutions. Turbidity was noted to be decreased in the reading of optical density compared with control by a microplate reader. The ANOVA single-factor analysis between the methanol and oil MIC values showed significant differences (*P* value < 0.05), with F > F critical, where *P* value = 0.00, F = 3779.233, and F critical = 7.7. As shown in Table 3, Figs. 11, 12.

**Table 3:** *Moringa oleifera* extracts against *Mycobacterium* tuberculosis variant *bovis.*

Isolate number	Inhibition			
	Methanolic leaves extract		Oil seed extract	
	Sensitivity (µg/ml)	MIC* (µg/ml)	Sensitivity % (v/v)	MIC* % (v/v)
1	1000	7.8	100	50
2	3000	32	100	50
3	1000	30	100	50

*Significant, *P* value = 0.00 (*P* value < 0.05).

### In silico docking prediction mode of action potential of *Moringa oleifera* anti-mycobacterial

**Fig. 13:**
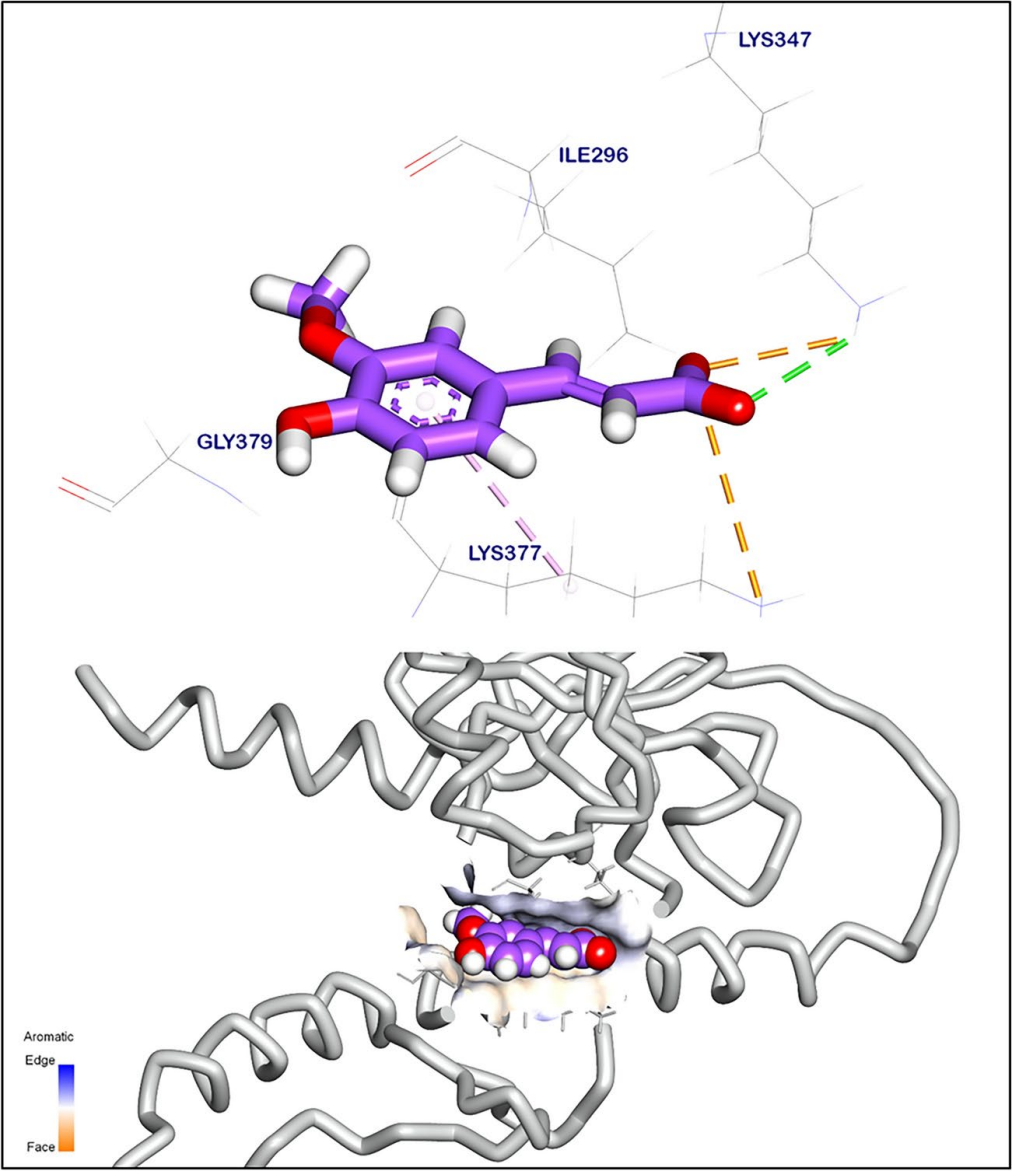
3D orientation (upper) and surface mapping (lower) of ferulic acid against ribosomal protein S1. Hydrogen interactions are presented with a green line, and the pi interactions are shown in purple lines. The software used to create the images was Biovia Discovery Studio Visualiser 2024. https://discover.3ds.com/discovery-studio-visualizer-download.

The binding mode of ferulic acid to ribosomal protein S1 exhibited ∆G equal to − 5.95 kcal mol^−1^. It formed a hydrogen bond with lysine (Lys347) at 2.72 Å. In addition, it formed one hydrophobic π-interaction and another two ionic bonds with Lys377 and Lys347 (Fig. 13).

**Fig. 14:**
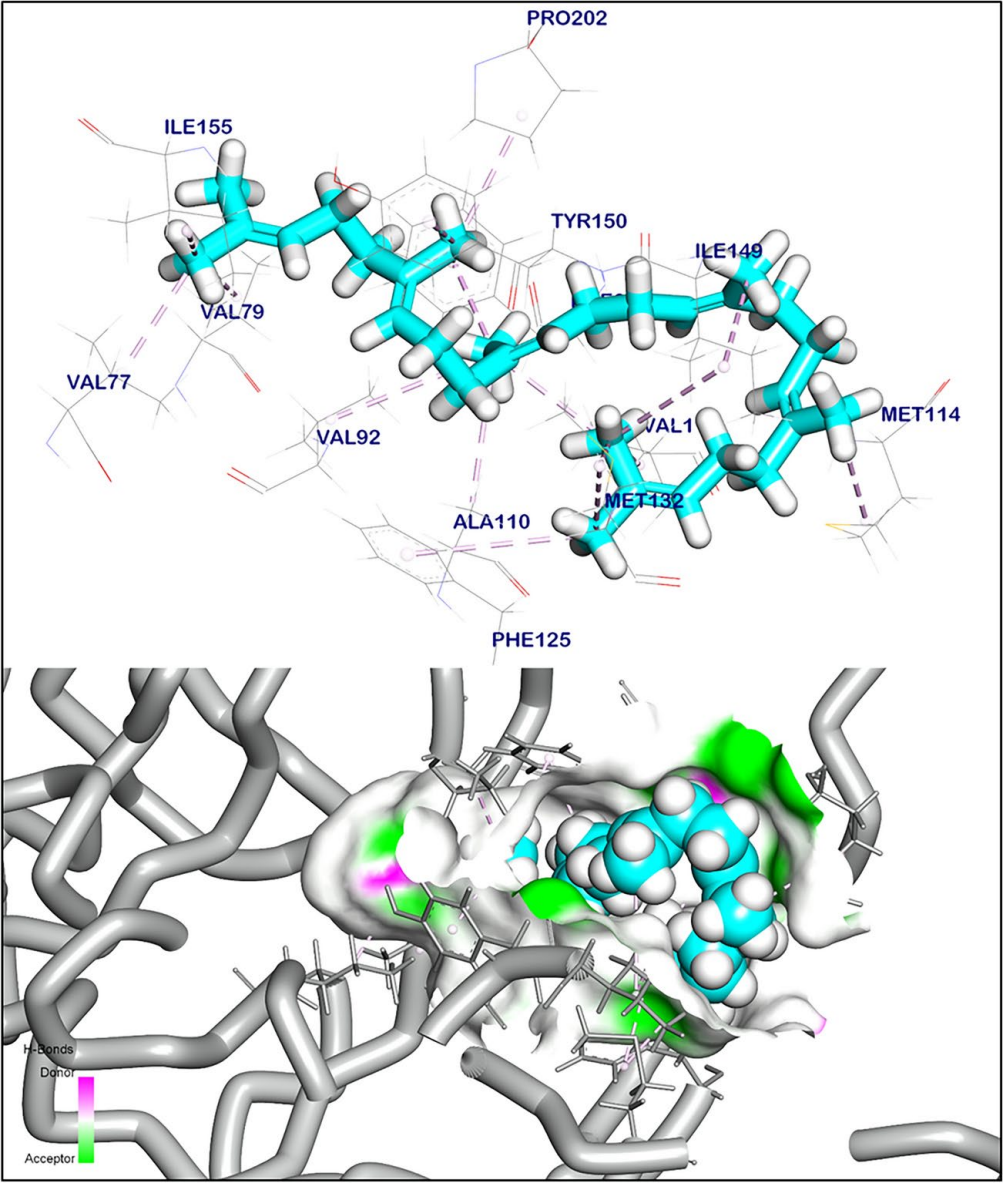
Orientation (upper) and surface mapping (lower) of squalene against lipoprotein LprF, pi interactions are shown in purple lines. The software used to create the images was Biovia Discovery Studio Visualiser 2024. https://discover.3ds.com/discovery-studio-visualizer-download.

The binding mode of squalene to lipoprotein LprF exhibited ∆G equal to − 6.11 kcal mol^−1^. Squalene showed seventeen hydrophobic π-interactions with methionine (Met 114), isoleucine (Ile 149), phenylalanine (Phe 125), Met 132, valine (Val 112), Val 79, Ile 155, Val 77, proline (Pro202), Val92, tyrosine (Tyr150), alanine (Ala110), and Phe 89 (Fig. 14, Table 4).

**Table 4:** Binding energy (DG) and root-mean-square deviation (RMSD) of atomic positions interactions kilocalorie/mol (kcal/mole) of (Ferulic acid and squalene) against *Mycobacterium tuberculosis* variant *bovis* target sites (ribosomal protein S1 and lipoprotein LprF), respectively.

Tested compounds	RMSD value (Å)	Docking (Affinity) score (kcal/mol)	Interactions	
			Hydrogen Bond (HB)	Pi-interaction
Ferulic acid	1.11	− 5.95	1	1
Squalene	1.27	− 6.11	0	17

## Discussion

Tuberculosis is a continuous public health risk and represents its indexes^48^. As recorded by the World Health Organization, the maximum rate of infection and death is recorded in low incoming and developing countries^49^. It is considered the ninth cause of death worldwide and the main cause of death due to single infectious cells^50^. *Mycobacterium bovis*, a member of the *M. tuberculosis* complex, is well known for causing bovine tuberculosis and is an etiological agent of human tuberculosis^51^. *Mycobacterium* is a zoonotic and infectious pathogen to humans and animals^52^.

Animal tuberculosis is caused by *M. bovis,* which infects a wide range of wild and domestic mammals^53^. It has significant economic importance because it affects productivity, morbidity, and mortality. Animal *Mycobacteria* are diagnosed either antemortem by assessing cellular immunoreactivity or postmortem by investigating tissue histology, cultivating the microbe, and using the PCR technique for molecular diagnosis^54^. The *Mycobacterium* culturing process is not easy. It represents the main successful character for *Mycobacterium* diagnosis^55,56^.

This study recorded *Mycobacterium* isolation from camels that were imported from southern countries lining Egypt. The sequencing results support the global spread of Mycobacteria and its presence in various species, including humans, large animals, and pets, indicating the need for heightened attention to infection control and safety processes. The current study identified four isolates confirmed as *M. bovis* through conventional PCR and Sanger sequencing of the mbp70 gene. These isolates exhibited 100% identity with CP130810 (Omani tuberculosis), CP139557 (novel *M. bovis* isolated from a cat in Ireland), CP158172 (human sputum from Thailand), CP1497080 (human isolates from Congo), and OZ023108 (Switzerland). CP133602 is a mutant *M. tuberculosis* variant *bovis* isolate (PHOPR KO3) from cattle in Ireland. CP133660 is *Mycobacterium orygis* isolated from cattle lung tissue in Kolkata, India.

The present study encourages the application of molecular epidemiology^57^, which has facilitated a better understanding of the global phylogeography and the extent of multidrug-resistant *Mycobacterium*. It performs a vital role in understanding the transmission dynamics of drug-resistant TB across Africa, and addressing these key knowledge gaps will guide effective TB treatment in high-risk population groups. Additional studies are required to better understand the epidemiology and associated factors of drug-resistant TB in Africa as a whole^58–61^.

Unfortunately, the unbiased characterization by Mi-seq Illumina for two selected isolates declared that the abundance gene sequence data belonged to *Micromonosporaceae,* and deep sequencing was unclear. We should consider that this may explain the results from Mi-seq Illumina, which indicated a tendency toward *Actinobacteria* and *Micromonosporaceae*.

An extraordinarily evolutionary envelope relationship between *Actinobacteria*, *Corynebacteriales,* and *Micromonospora* has been assessed by Vincent et al.^62^. *Mycobacterium* belonged to the phylum *Actinomycetota*^63^. This genus has cell walls with a waxy lipid-rich outer layer that contains high concentrations of mycolic acid^64^. *Actinomycetota* or *Actinobacteria* are a diverse phylum of Gram-positive bacteria with high GC content.

*Mycobacterium tuberculosis* and *M. bovis* are still a good example of interspecific evolutionary relationships as well as intraspecific evolution and their spread across the world^65^. Previous studies hypothesized that *M. bovis* and *M. tuberculosis* represented the same branch or lineage within the *Mycobacteriaceae*. In this situation, *M. tuberculosis* originated from *M. bovis,* and the bacteria had been transmitted to man from domesticated animals in a zoonotic scenario^66^. In *M. bovis*, studies indicate a much shallower level of evolution with a much more recent ancestry of the pathogen. *Mycobacterium bovis* is primarily found in Africa and, similar to *M. tuberculosis*, is believed to have an African origin or a nearby one, such as in Southwestern Asia. Two studies published in 2020 suggested that the spread of *M. bovis* in Africa started in Eastern Africa^67,68^.

However, next-generation sequencing (NGS) methods suggest high throughput rewards, and Sanger sequencing maintains significance for validating NGS outcomes due to its recognized accuracy^69^. The crucial situation is that metagenomic data indicate the potential presence of multiple pathogens; track-up assays should be posed to target detected pathogen sequences after conventional PCR, alongside predictable diagnostic methods, containing isolation and characterization. It provided further proof of a disease relationship^27^. The amount of sequence variation may be inadequate for species-level identification, and as a result, classification, in some cases, may be possible at the family or genus level^70,71^.

Histopathology examination of lung tissues and lymph nodes represented the presence of unspecific lesions accompanied by lymphocytes and neutrophils through their focal replacement, consequently, thickening of interalveolar septae. It might suggest that camel immunity differs from other animals; thus, the absence of a typical granulomatous reaction appearance requires an extremely late stage of the disease. The current report about the absence of formal granuloma in dissected camel-infected lung and lymph node tissues with *Mycobacterium* may supported by Palmer et al.^72^, who proved the occurrence of early pulmonary lesions in cattle infected with *M. bovis* through aerosolized experiments. They declared that understanding host–pathogen interactions at the granuloma level is critical; it requires more advanced interleukin-cytokines studies. Moreover, tuberculosis was diagnosed in the patient and was not confirmed with pathology^73^.

The effect of *Moringa oliefera* against *Mycobacterium* in camel has not been issued before. In this study, the application of *Moringa oliefera* on isolates by tetrazolium technique revealed that MIC ranged from 7.8 to 32 µg/ml by methanol leaves extract and 50% (v/v) by this seed oil. Tetrazolium microplate assay is a great reproducible method for evaluating mycobacterial growth^74^. Several studies used *M. oleifera* to detect the antibacterial activity of different pathogens^75^. It affected some pyogenic bacteria^76^.

The current results recommended the reproducible effect of *Moringa* oil. Seven essential oils and potential hydrocarbons were identified; oleic acid retains a higher percentage of 62.45%. Of the few studies on *M. oleifera* concerning its effect on Mycobacteriae, some studies examined the chemical composition of seed oil. None of the studies reported squalene and androstane derivatives^77,78^. Hexadecanoic and oleic acids are the primary components of any plant-derived oil^79,80^. Although several reports indicate the antibacterial effect of oleic acid^81^, detected the antitubercular activity of *M. oleifera* oil on a clinical human isolate, finding an effect at 25 µg/ml, which may support our results^82^. This study explained that the results depended on the high concentration of oleic acid content in the analyzed oil (58.88%). Moreover, they assessed that the mechanism had not been understood.

We have a different opinion in this situation, and the appearance of the squalene hydrocarbon peaks in this study caught our attention and might have a potential role in *M. oleifera* oil antimycobacteria. However, this might be supported through an in-silico prediction study. The in-silico surrender of lipoprotein LprF of *M. bovis* to squalene results from the unexpected formation of seventeen hydrophobic π-interactions with 13 distinct amino acids. Besides the congruency effect of ferulic acids through lysine hydrogen bonds, one hydrophobic π interaction and another two ionic bonds. These events support the potential anti-mycobacterium effect of both *Moringa oliefera* extracts, which overcame the virulent effect of waxy layer mycolic acid, representing the key role in drug resistance and anti-mycobacterial agents.

These findings are supported by the fact that waxy layers of *Mycobacterium* cell wall due to the presence of peptidoglycan, arabinogalactan, and mycolic acid is known as mycolylarabinogalactan–peptidoglycan complex, which imparts specific properties that enable this pathogen to resist chemicals, anti-mycobacterium drugs, and the host's immune system^83,84^.

Squalene is a natural dehydro-tri-terpenic hydrocarbon (C30H50) with six double bonds, known as an intermediate in the biosynthesis of phytosterol in plants or cholesterol in animals. The most famous animal source of squalene is shark liver oil, which represents the richest natural source. Since ancient eras, anglers all over the world have benefited from the important properties of oil, such as its ability to treat wounds or several circumstances of respiratory tract infections. Opposite to the squalene of shark oil, vegetal origin is not human harm^85^.

Squalene is recognized in many vegetal oils in different concentrations. Vegetal-origin squalene is highly appreciated. The first plant oil was in olive oil^86^. Various concentrations of squalene have been estimated through gas chromatography by Frega et al.^87^ in diverse types of oil like olive, soybean, and corn. The effect of squalene derived from *Rhus taitensis* on *M. tuberculosis* was studied^88^. Squalene reduces the effects of chemotherapy and is considered a chemo-protective effect. It facilitates vaccine delivery and has antioxidant effects^89^.

The ribosomal protein S1 has been known for its role in *M. tuberculosis* drug resistance^90,91^. The current hypothesis about anti-mycobacterial inhibition was rendered due to *Moringa oliefera* leaves containing ferulic acid that docked professionally with *Mycobacterium* ribosomal protein. As *M. tuberculosis* complex causes severe respiratory disease in animals and humans, some studies about *M. oleifera* leaf's components polyphenol compounds have confirmed the potential for anti-inflammatory and pneumonia treatment^92,93^.

The current finding about androstane components in *Moringa oliefera* oil focused on new strategies and further studies. Androstane is recognized as a C19 steroidal hydrocarbon with a gonane core. Androstane can exist as either of two isomers, 5α-androstane and 5β-androstane^94^. Androstenedione is produced in male and female gonads and the adrenal glands, and it has been identified for its crucial role in the manufacture of estrogen and testosterone^95^.

The role of anti-mycobacterial derived from natural plants, especially *Moringa oliefera* extracts, has not been discussed before. This study considered a novel prevention protocol and treatments for TB by new therapeutic candidates. It might help camel breeders protect and care for the animal and its caregivers. It will help the public health decision-maker to increase awareness among the general residents regarding *Mycobacterium*. This issue opens a new field for pharmaceutical companies interested in new research to combat tuberculosis.

This study might have a limitation as the in vivo application might contribute some keys to the mode of action; thus, we will try a challenge in the prospective study.

## Conclusion

To conclude, camels are a hazardous factor in tuberculosis prevalence and should be considered. *Mycobacterium tuberculosis* variant *bovis* has a public health and zoonotic impact because of veterinary practices conducted with camels. *Mycobacterium* diagnosis on the genetic level is particularly important for epidemiology studies. This study contributes to solution evaluation requirements that might be done for the current situation; however, it serves as a potential solution for tuberculosis. Finding natural solutions to eliminate *M. bovis* would be welcome in light of the current epidemiology and multidrug resistance conditions to increase the immunity of camels, workers, and veterinarians. *Moringa oleifera* leaves might be a prospective protective feed additive for camels. Oil might be recommended for treatment or defense against human tuberculosis.

## Data Availability

The datasets originated from Sanger sequencing analyzed during the current study are available at (https://blast.ncbi.nlm.nih.gov/Blast.cgi) named DRC-EG-CAMEL accession number PQ036932.

## Abbreviations

TB: Tuberculosis
NGS: Next-generation sequencing
HPC: Hexadecylpryridinium Chloride Monohydrate
TBE buffer: Tris–Borate-EDTA buffer
MIC: Minimum inhibition concentration
TEMA: Tetrazolium microplate assay
V/cm: Volt per centimeter
HPLC: High-performance liquid chromatography
GC-MS: Gas chromatography-mass spectrometry
UV, MMFF: Ultraviolet, Merck molecular force field
Nm–m–mm: Nanometer–Millipore–millimeter
mg/ml, µg/ml: Milligram/milliliter, microgram/milliliter
DNA–PCR: Deoxyribonucleic acid–Polymerase Chain Reaction
F–R–bp: Forward–Reverse–Base pair
BLAST: Basic local alignment search tool
OTU: Operational taxonomy unit
ANOVA: Analysis of variance
*P* value- F crit: Probability value- F critical
PDB: The protein data code
Lys: Lysine
Met: Methionine
Ile: Isoleucine
Phe: Phenylalanine
Pro: Proline
Val: Valine
Tyr: Tyrosine
Ala: Alanine
DG-RMSD: Binding energy-root-mean-square deviation
kcal/mol: Kilocalorie/mole
NCBI: The National Center for Biotechnology Information
LprF: The lipoprotein
DRC-EG: Desert Research Center-Egypt
